# Highly Efficient Purification of Recombinant VSV-∆G-Spike Vaccine against SARS-CoV-2 by Flow-Through Chromatography

**DOI:** 10.3390/biotech10040022

**Published:** 2021-10-12

**Authors:** Elad Lerer, Ziv Oren, Yaron Kafri, Yaakov Adar, Einat Toister, Lilach Cherry, Edith Lupu, Arik Monash, Rona Levy, Eyal Dor, Eyal Epstein, Lilach Levin, Meni Girshengorn, Niva Natan, Ran Zichel, Arik Makovitzki

**Affiliations:** Department of Biotechnology, Israel Institute for Biological Research (IIBR), Ness-Ziona 74100, Israel; zivo@iibr.gov.il (Z.O.); yaronk@iibr.gov.il (Y.K.); yaakova@iibr.gov.il (Y.A.); einatt@iibr.gov.il (E.T.); lilachc@iibr.gov.il (L.C.); edithl@iibr.gov.il (E.L.); arikmo@iibr.gov.il (A.M.); ronal@iibr.gov.il (R.L.); eyalo@iibr.gov.il (E.D.); eyale@iibr.gov.il (E.E.); lilachl@iibr.gov.il (L.L.); menig@iibr.gov.il (M.G.); nivan@iibr.gov.il (N.N.); ranz@iibr.gov.il (R.Z.)

**Keywords:** rVSV, SARS-CoV-2, downstream process, chromatography, membrane adsorbers

## Abstract

This study reports a highly efficient, rapid one-step purification process for the production of the recombinant vesicular stomatitis virus-based vaccine, rVSV-∆G-spike (rVSV-S), recently developed by the Israel Institute for Biological Research (IIBR) for the prevention of COVID-19. Several purification strategies are evaluated using a variety of chromatography methods, including membrane adsorbers and packed-bed ion-exchange chromatography. Cell harvest is initially treated with endonuclease, clarified, and further concentrated by ultrafiltration before chromatography purification. The use of anion-exchange chromatography in all forms results in strong binding of the virus to the media, necessitating a high salt concentration for elution. The large virus and spike protein binds very strongly to the high surface area of the membrane adsorbents, resulting in poor virus recovery (<15%), while the use of packed-bed chromatography, where the surface area is smaller, achieves better recovery (up to 33%). Finally, a highly efficient chromatography purification process with Capto^TM^ Core 700 resin, which does not require binding and the elution of the virus, is described. rVSV-S cannot enter the inner pores of the resin and is collected in the flow-through eluent. Purification of the rVSV-S virus with Capto^TM^ Core 700 resulted in viral infectivity above 85% for this step, with the efficient removal of host cell proteins, consistent with regulatory requirements. Similar results were obtained without an initial ultrafiltration step.

## 1. Introduction

The novel coronavirus SARS-CoV-2 is the causative agent of the COVID-19 respiratory disease, which to date has infected over 220 million people and killed over 4.6 million [1,2], initiating a global economic and social crisis [3,4]. The SARS-CoV-2 virus is an enveloped single-stranded positive sense RNA virus that enters host cells by mediation of a transmembrane spike (S) glycoprotein binding to an angiotensin-converting enzyme (ACE2) [5]. The S protein comprises two functional subunits, the S1 subunit responsible for binding to the host cell receptor and the S2 subunit responsible for the fusion of the viral and cellular membranes [6]. Entry of the coronavirus into the cells is a complex process that requires proteolytic cleavage of the S protein, promoting virus–cell fusion and entry [7].

Over 300 vaccines are being developed against the disease, of which, to date, approximately 110 are under clinical evaluation [8], including vaccines listed by the WHO for emergency use [9] and vaccines authorized for emergency use by the FDA [10]. The vaccines developed encompass a large range of technology platforms, including nucleic acid (DNA and RNA), viral vectored vaccines, recombinant protein, and live attenuated/inactivated approaches [11].

Vesicular stomatitis virus (VSV), a negative-stranded RNA virus that causes a disease in animals but rarely in humans [12], has been utilized as a potential viral vector vaccine [13,14,15,16], such as the recently approved Ebola vaccine [17]. VSV has a rigid “bullet” shape, and the virion has a lipid envelope decorated with glycoprotein (G) spikes that encloses a nucleocapsid composed of RNA plus nucleoprotein (N) and an associated matrix formed by (M) proteins [18]. Intact virions are approximately 70 nm in diameter and 180 nm long [19]. An rVSV-based vaccine (rVSV-∆G-spike) was recently developed by the Israel Institute for Biological Research (IIBR) [20], in which the VSV-G protein was replaced with the spike protein of the SARS-CoV-2 virus (SARS-CoV-2-S), creating a recombinant replicating virus. The cDNA vector is created and encodes the spike of SARS-CoV-2 expressed on the viral membrane. A single-dose vaccination of hamsters with rVSV-SARS-CoV-2-S (rVSV-S) elicited a safe and effective response against the SARS-CoV-2 challenge [20], and the vaccine is currently being evaluated for safety and potential efficacy in Phase I/II clinical trials [21]. In addition, a recent study generated a high-titer, replication-competent chimeric VSV expressing the SARS-CoV-2 S protein that performs similarly to a SARS-CoV-2 clinical isolate across multiple neutralization tests [22].

Development of the rVSV-S vaccine requires a downstream process (DSP) for purification of the vaccine substance. The aim of the downstream process is to eliminate contaminants, such as host cell proteins (HCPs), DNA, and other impurities from the cell culture media to levels that meet regulatory requirements. Residual HCPs have the potential to affect product quality, safety, and efficacy, while the risks associated with residual DNA are infectivity and oncogenicity [23]. The purification process must remove as many HCPs as feasible to make the product as pure as possible [24], ultimately obtaining a product with high purity and potency [25].

The DSP of cell culture-derived viral vaccines involves two discrete operations. The initial purification entails endonuclease digestion of the harvested supernatant, clarification, and concentration of the residual viral vector [26]. The final purification step uses chromatography to meet purity requirements [27,28]. In contrast to classical laboratory methods for purifying viral vectors (i.e., density gradient centrifugation), chromatography is easily scaled up with high capacity and is widely used in the purification of viral vectors and vaccines [29]. In addition, adsorption methods offer important advantages such as the use of high flow rates and preservation of labile viruses typically using mild conditions (i.e., low pressure and shear forces) to separate the virus through the chromatographic matrix [29].

Purification of rVSV by chromatography, specifically by packed-bed anion-exchange chromatography, has been previously described [30]; however, the efficacy of HCP removal was not reported. In the patent literature, there are reports of rVSV purification using membrane adsorption chromatography [31] and purification schemes composed only of filtration methods [32]. Chromatography is typically performed by packed-beds, membrane adsorbers, and monoliths, implemented in size exclusion, ion exchange, affinity, hydrophobic interaction, and mixed-mode chromatography [27].

In the past decade, membrane adsorbers have emerged as a cost-effective chromatography technique [33], specifically for their ability to purify large biomolecules such as viruses and process large volumes [34,35,36]. Ion-exchange membranes have been reported to have high dynamic binding capacity and are implemented in many virus purification applications, such as lentiviral vectors [37,38], the recombinant baculovirus [39], the influenza A virus [40], the adeno-associated virus [41], rotavirus-like particles [42], and the modified Vaccinia Ankara (MVA) virus [43]. Similar to membrane adsorbers, monolithic chromatographic supports are characterized by hollow structures with high porosity, providing a large surface area, high binding capacity, and high flow rates [44], thus are particularly applicable in processing large biomolecules such as virus particles [45,46]. Reports of virus purification using anion-exchange monoliths include baculoviruses [47], influenza virus A and B subtypes [48], HIV-1 gag VLPs [49], and the vaccinia virus [50].

Mixed mode (multimodal) chromatography (MMC), in which solutes interact with stationary phase through more than one interaction mode or mechanism, is becoming increasingly popular in biopharmaceutical applications [51]. To this end, the Capto^TM^ Core 700 (CC700) resin manufactured by Cytiva, combining size exclusion (molecular weight cutoff of 700 kDa) and ion-exchange chromatography, is suitable for the separation of large molecules, such as viruses collected in the flow-through, while smaller impurities enter and bind to the positively charged octylamine ligand. Chromatography using CC700 has been shown to be effective in purifying influenza viruses A and B [52,53], AdVs [54], the hepatitis C virus [55], and the respiratory syncytial virus [56].

This study compares several purification strategies of rVSV-S using the aforementioned chromatography methods, including membrane adsorbers and packed-bed ion-exchange chromatography. Finally, a highly efficient chromatography purification process with CC700 resin is described, achieving high HCP removal, as well as high yields and potency. The findings, reported here for the first time, set the foundation for a highly efficient downstream purification scheme for the production of the rVSV-S vaccine.

## 2. Materials and Methods

### 2.1. Cell Culture and Virus Culture

The rVSV-S virus was replicated in Vero cells (WHO Vero RCB 10–87) and widely accepted by the WHO and regulatory agencies for the manufacturing of human viral vaccines [57] using serum-free medium (SFM) (FLEX20, Biological Industries, Beit Haemek Israel). Vero cells were seeded into 6342 cm^2^ multi-trays (MTs) (NUNC EasyFill Cell Factory System, Thermo Fisher Scientific, MA, USA cat. # 140400) at an initial density of 1,500,001 cells/cm^2^ and a total volume of 1000 mL SFM FLEX20 medium. Typically, 3 to 6 MTs were grown in parallel, resulting in a working volume of 3 to 6 L. Cells were grown to confluence in MT at 37 °C and 5% CO_2_ and then infected with an estimated 0.1 multiplicities of infection (MOI) of rVSV-S. The inoculated cells were maintained for 72 h in MT at 37 °C and 5% CO_2_ and harvested into 3–6 one-liter flasks.

### 2.2. Denarase Treatment and Clarification

Harvest was subjected to endonuclease digestion using Denarase endonuclease (20804-5M, GMP Grade, c-LEcta, Leipzig, Germany). Digestion was performed with 60 U/mL endonuclease in the presence of 2 mM MgCl_2_ (MA110, Spectrum) for 3 h in a shaker incubator at 37 °C under constant and mild agitation. The digested viral harvested supernatant was clarified by a filter train with pore sizes of 3 μm (Sartopure PP3, size 4, 0.018 m^2^, polypropylene, Sartorius) 1.2 μm (Sartopure PP3, size 4, 0.013 m^2^, polypropylene, Sartorius) and 0.45/0.2 μm (Sartopore platinum, size 4, 0.021 m^2^, PES, Sartorius).

### 2.3. Ultrafiltration

Ultrafiltration was performed using a 750 kDa nominal molecular weight cutoff (NMWC) with 1 mm diameter polyethersulfone (PES) hollow fiber (HF) membrane cartridges (Cytiva, Marlborough, MA, USA) on a commercial ultrafiltration system (KRONOS, Solaris Biotechnology, Mantova, Italy) at 9 ± 2 °C. The endonuclease-digested and clarified cell culture harvested supernatant was concentrated 3–4-fold by volume and diafiltrated with five diafiltration volumes against the equilibration buffer (100 mM NaCl in 20 mM Tris-HCl, pH = 7.2, and 4% D-trehalose (Pfanstiehl, Waukegan, IL, USA)).

### 2.4. Chromatography Conditions and Resins

Chromatography experiments with packed-bed chromatography resins and membrane adsorbers were performed using an AKTA AVANT 25 (Cytiva, Marlborough, MA, USA) with UNICORN software. The flow rate used in all packed-bed chromatography experiments was 1 mL/min, according to the recommended flow rate by the manufacturer. The following strong anion exchange chromatography resins were used in this study. QX-L (HiTrap 1 mL column) was supplied by Cytvia and Fractogel^®^. EMD TMAE Hicap supplied by Merck (Boston, MA, USA) with a particle size of 48–60 µm. The weak anion exchanger resin used in this study was Fractogel^®^ EMD DMEA supplied by Merck (Boston, MA, USA) with a particle size of 48–60 µm. Equilibration buffer comprised 20 mM Tris, 4% trehalose, pH = 7.2, and varying amounts of NaCl (50, 100, 150 mM) depending on the experiment performed. Elution was performed by a 20 min gradient with 2 M NaCl, 20 mM Tris, and 4% trehalose, pH = 7.2; fractions of 1 mL were collected.

### 2.5. Membrane Adsorbers

Four different membrane adsorbers were used in the bind-elute mode. A Mustang^®^ Q anion exchange membrane chromatography with a packed-bed of 5 mL (PALL, Ann Arbor, MI, USA). A 0.2 mL Natrix^®^ Q strong anion exchange chromatography membrane was supplied by Merck (Boston, MA, USA). QF5 was the strongly basic anion exchange membrane chromatography with a 0.14 mL bed volume (Sartorius, Goettingen, Germany) and DF5 was the weakly basic anion exchange membrane chromatography with a 0.14 mL bed volume (Sartorius, Goettingen, Germany). The flow rate used in all membrane chromatography experiments was 5 mL/min, according to the recommended flow rate by the manufacturer. Membranes were equilibrated with 150 mM NaCl, 20 mM Tris, and 4% trehalose, pH = 7.2. Elution was performed using a 20 min gradient with 2 M NaCl, 20 mM Tris, and 4% trehalose, pH = 7.2.

### 2.6. Purification Using Capto^TM^ Core 700 Resin

A HiTrap CC700 1 mL prepacked multimodal chromatography column (Cytiva, Marlborough, MA, USA) and HiScale 26/20 packed with 100 mL CC700 resin were equilibrated with 10 CV (20 mM Tris, 4% trehalose, 150 mM NaCl, pH = 7.2). The flow rate with the HiTrap 1 mL column was 1 mL/min, and that for the 100 mL column was 20 mL/min. The flow-through was collected, and impurities were eluted using 10 CV of 2 M NaCl and 20 mM Tris, pH = 7.2.

### 2.7. Determination of Binding Capacity of Vero HCP

The binding capacity of Vero HCP for the CC700 column was determined by loading the supernatant containing rVSV-S virus directly to the column, following endonuclease digestion and initial clarification. The material was loaded onto a 1 mL column at a flow rate of 1 mL/min, and fractions of 2 mL were collected. The breakthrough (DBC_1%_-more than 1% of the feed concentration found in the flow-through fractions) was determined by quantifying the HCP content in all fractions collected.

### 2.8. Analytical Methods

#### 2.8.1. Infectivity Assay

The infectivity assay was performed as previously described [20]. Vero E6 cells were seeded in 6-well plates (7 × 10^5^ cells/well) and grown overnight in growth medium. Serial dilutions of rVSV-S were prepared in minimal essential medium (MEM) and used to infect Vero E6 monolayers in duplicate (200 µL/well). Plates were incubated for 1 h at 37 °C and 5%-CO_2_ to allow the virus to penetrate the cells. Then, 2 mL/well of overlay [MEM X 2 containing 0.4% tragacanth (Merck, Israel)] was added to each well, and the plates were incubated at 37 °C and 5%-CO_2_ for 72 h. The media were then aspirated, and the cells were fixed and stained with 1 mL/well of 0.1% crystal violet solution (Biological Industries, Beit Haemek, Israel). The number of plaques in each well was determined, and the rVSV-S titer was calculated. Each assay included a positive control plate containing 6 replicates (wells) of a single, known sample dilution and 2 negative control wells with MEM that did not contain the virus. The titer in plaques forming units (PFU)/mL for each of the samples is the average of the values calculated from the tested dilutions.

#### 2.8.2. Host Cell Protein Quantification Assay

The level of residual proteins in Vero cells was measured using a Vero host cell protein (HCP) ELISA kit (Cygnus Technologies, Southport, NC, USA) following the manufacturer’s instructions. A total amount of 50 µL of diluted samples was added to microtiter strips coated with an affinity-purified capture goat polyclonal anti-Vero cell antibody. The horseradish peroxidase (HRP) enzyme-labeled anti-Vero cell antibody (goat polyclonal, 100 µL) was added to the wells and incubated for 2 h at 25 °C with shaking at 500 rpm. The wells were washed 4 times with wash buffer to remove any unbound reactants. Then, 100 µL of 3,3′,5,5′ tetramethylbenzidine (TMB) solution was immediately added to the plate and incubated at 25 °C for 30 min. The reaction was stopped using 100 µL of 0.5 M H_2_SO_4_ stop solution, and the absorbance was read at 450 nm using a CLARIOstar multimode reader (BMG LABTECH, Ortenberg, Germany). All standards, controls, and samples were assayed in duplicate; 2 different dilutions of the sample were tested. The residual Vero HCP concentration (ng/mL) in a tested sample was calculated by interpolation from a 4-parameter nonlinear fit regression of the standard curve after subtracting the blank.

#### 2.8.3. Residual DNA Measurement

The concentration of Vero cell residual DNA was determined using quantitative PCR (qPCR) with a resDNASEQ™ Quantitative Vero DNA Kit, cat# A41797 (Applied Biosystems™-Thermo Fisher Scientific, Waltham, MA, USA), based on TaqMan real-time qPCR technology. The assay kit contained concentrated reagents and buffers, including DNA dilution buffer (DDB), control Vero DNA, water, primers, and DNA polymerase. Genomic DNA of the tested (unknown) samples was first isolated and recovered with magnetic beads using a PrepSEQ Residual DNA Sample Preparation Kit, cat# 4413686 (Applied Biosystems™-Thermo Fisher Scientific). A DNA standard curve was prepared by serial 10× dilutions of the control Vero DNA. All samples are diluted in DDB. A reaction mix was prepared according to the kit’s instructions and added to wells in a white 96-well plate (20 µL/well, in duplicate). Then, DNA samples (standards or unknowns) were added (10 µL/well), and the plate was transferred to a LightCycler^®^ 96 Instrument (Roche Life Science, Penzberg, Germany). At the end of the reaction, the DNA concentration in the tested sample was back-calculated by interpolation from a linear regression fit of the standard curve.

## 3. Results

### 3.1. Clarification and Ultrafiltration

The harvest solution was treated with nuclease, and a reduction of 99.9% in DNA concentration was achieved [26]. Host cell debris, large aggregates, and insoluble contaminants were clarified using a filter train composed of 3 µm and 1.2 µm inert polypropylene depth filters and a 0.45/0.2 µm membrane filter. Throughout the clarification process, approximately 85% viral recovery was attained. Ultrafiltration and 4–5-fold diafiltration were performed with a 750 kDa HF against equilibration buffer, resulting in virus recovery of 45–55% [26]. The composition of the buffers used in this report, namely Tris and D-trehalose, is based on their importance as stabilizers in biological therapeutic products [58,59], specifically for the production of rVSV [30,60]. Unless otherwise specified, the concentrated samples following the ultrafiltration step were the starting material of all chromatography experiments described ahead. As DNA clearance with nuclease treatment met regulatory requirements (less than 10 ng host cell DNA per dose), it was not determined in the following chromatography experiments.

### 3.2. Virus Stability

Viral stability was evaluated during chromatographic ion-exchange separation using a Q-XL column by supplementing 2% or 4% sucrose or trehalose to the equilibration and elution buffer. Sucrose or trehalose was added to the concentrated samples following the UF step. The samples were then loaded to a 1 mL Q-XL column at 1 mL/min, and elution was performed with 2 M NaCl buffer including the same sugar additive at the same concentration. Following each run, virus titer was determined. Supplementation with 2% and 4% sucrose resulted in 26% and 23% viral recovery, respectively. Supplementation with 2% and 4% trehalose resulted in 16% and 33% recovery, respectively; thus, the addition of 4% trehalose was used in the equilibration buffer of all chromatographic runs performed in this report (see Section 3.3).

### 3.3. Membrane Adsorbers

We initially chose four membrane adsorbers and determined their ability to purify the rVSV-S virus. All experiments were performed using membrane anion exchangers in the bind-elute mode, where the virus was allowed to bind the membrane and was further eluted in a gradient of 0–2 M NaCl. The total recovery of all membrane adsorbers tested was low (<15%). Table 1 summarizes virus recovery for all experiments performed in this report. The Mustang^®^ Q and Natrix^®^ Q membrane adsorbers showed the highest virus recovery (13% and 14%, respectively), while the QF5 and DF5 membrane adsorbers demonstrated very poor recovery (<5%). Additionally, we tested a monolith column (CIMac^TM^ QA); however, the virus was not recovered. As virus recovery was low, the results did not justify further exploration of this technique; thus, HCP clearance was not determined, and packed-bed chromatography was evaluated.

### 3.4. Packed-Bed Ion-Exchange Chromatography

**Figure 1:**
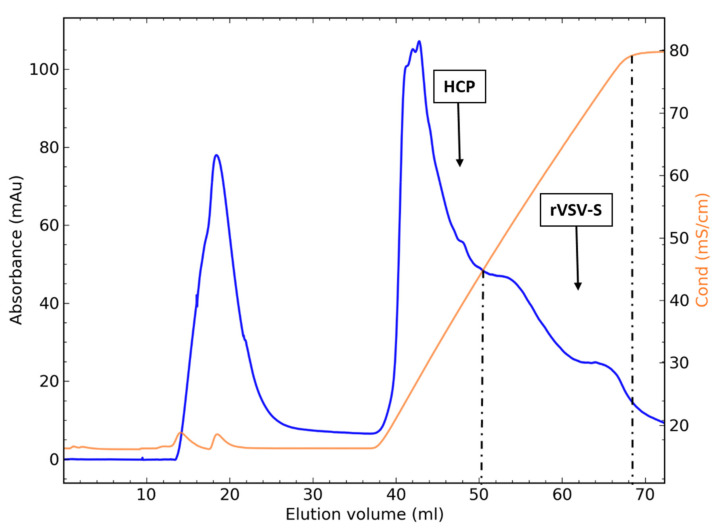
Chromatogram of rVSV-S purification on a Q-XL ion exchanger with 150 mM NaCl. HCPs are eluted up to 45 ms/cm, and the virus is eluted between 45 and 80 ms/cm.

A comparison of strong and weak anion exchangers was performed by screening for virus recovery using different concentrations of NaCl (50, 100, and 150 mM) in an equilibration and elution buffer (Table 1). In all packed-bed chromatography experiments the virus bound to the chromatography media and was further eluted in the NaCl gradient. The use of 50 mM NaCl with the strong anion exchangers screened (Q-XL, Fractogel TMEA) resulted in very poor yields (<5%) compared to the weak anion exchanger (Fractogel DMEA) with 50 mM NaCl, which resulted in 27% recovery. The Fractogel TMEA strong ion exchanger achieved the lowest virus yields (18%). Higher NaCl concentrations (100 and 150 mM) increased the yields, specifically using the Q-XL column with 100 and 150 mM NaCl, which yielded the highest recoveries of 32% and 33%, respectively. As the Q-XL column achieved the highest virus recovery, it was examined more thoroughly to identify the conductivity range where the virus elutes from the column. Fractions throughout the 2 M NaCl gradient elution were collected and analyzed for virus infectivity and HCP clearance. Figure 1 shows a typical chromatogram using a Q-XL column. The results show (Table 2) that HCPs were eluted at conductivity levels between 16 and 45 mS/cm, while the virus was mostly eluted between conductivity levels of 45 and 80 ms/cm. Overall, a 33% recovery was achieved as well as 99% HCP clearance.

### 3.5. Capto^TM^ Core 700

We tested a mixed-mode CC700 resin in order to increase the virus recovery achieved by ion-exchange chromatography. The CC700 resin binds negatively charged molecules with a molecular weight that is smaller than 700 kDa, enabling the virus to pass through the column while capturing small negatively charged contaminants.

#### 3.5.1. Binding Capacity Determination of Vero HCP

Initially, the binding capacity of the CC700 column was determined by its ability to adsorb Vero HCP. Fifty CVs of supernatant containing rVSV-S virus (34.5 µg/mL HCP), following clarification, were directly loaded onto a 1 mL CC700 column at a flow rate of 1 mL/min. A total of 1.8 mg HCP was loaded onto the column, and a 1% breakthrough was reached following a load of 20 mL; thus, the capacity of the column for Vero HCP, yielding an overall HCP clearance of 99% (DBC_1%_), was determined to be 0.69 mg/mL.

#### 3.5.2. Flow-through Chromatography

**Figure 2:**
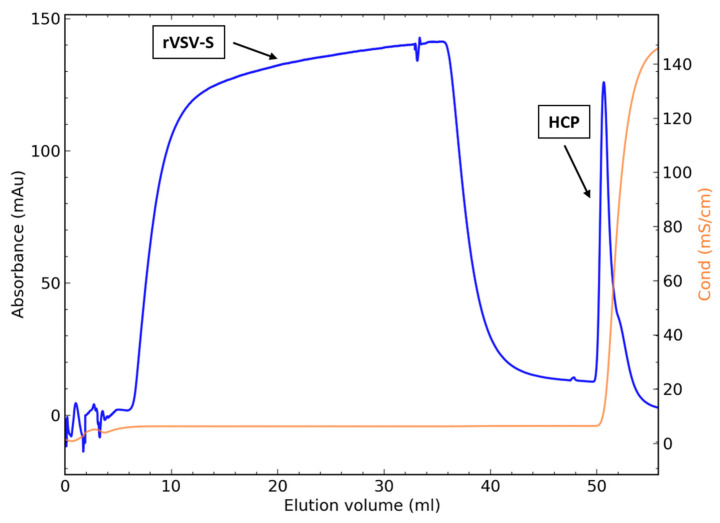
Chromatogram of a CC700 packed-bed 1 mL column. The flow-through was collected and analyzed for virus infectivity and HCP clearance.

Concentrated virus following the UF step including 100 mM NaCl was loaded and purified on a 1 mL CC700 column. Figure 2 depicts a typical chromatogram with the virus passing the column in the flow-through, while the HCPs are captured by the inner ligands of the CC700 resin and subsequently eluted in the 2 M NaCl regeneration peak. HCP levels in the flow-through reached overall low and acceptable levels (<250 ng/mL HCP).

#### 3.5.3. Direct Loading of the Clarified Harvest to Capto^TM^ Core 700

The possibility of directly applying the clarified harvest following the DNA cleavage step to the CC700 column without the initial ultrafiltration step was explored. To this end, 4% trehalose and 20 mM Tris were added to the enzyme-digested harvest and subsequently clarified, as described in the Methods section. Ten milliliters of the clarified harvest was directly loaded onto a 1 mL prepacked Cato Core 700 column, and the flow-through was collected. Viral recovery was 77%, and HCP (<100 ng/mL) reached regulatory demands. We further scaled up to a 100 mL column, where 1.4 L of the clarified harvest was directly loaded and purified on the 100 mL column packed with the CC700 resin, with similar results. The virus recovery was 75%, and residual HCPs were completely removed (<100 ng/mL).

## 4. Discussion

The objective of this work was to screen and evaluate chromatography methods for the purification of the rVSV-∆G-spike virus vaccine, recently developed by the Israel Institute for Biological Research [20]. Initially, purification using ion-exchange membrane chromatography was evaluated based on the net charge of proteins on the exterior of the viral capsid and their isoelectric point (pI) [61]. Membrane chromatography has been shaped to increase the available surface area for virus binding and has been reported to be used in a bind-elute mode as a capture step [62] or flow-through mode [63], with or without an additional polishing step, such as size exclusion chromatography [64]. In addition, membrane adsorbers enable the processing of large volumes of feed streams [63,64], which is advantageous when considering scale-up. A variety of anion-exchange membrane adsorbers were tested in capture mode, where the virus was eluted by a salt gradient. However, when applied, yields were very poor for all membrane adsorbers tested (<15% recovery). Moreover, no virus was recovered when purification was performed using a monolith column, similarly designed for purifying large macromolecular complexes such as viruses [45]. The poor virus recovery may be attributed to the large size of the virus (70 nm) and the spike protein, which binds positively and strongly to the large surface area of the membrane, subsequently decreasing the resolution and recovery with elution using a high salt concentration.

In the course of evaluating multiple conventional approaches for downstream purification (DSP) of vesicular stomatitis virus-based vaccine (rVSV-S), we determined that acceptable yields are obtained only with CC700 resin, a process that does not require the interaction of the large virus and large binding surface of the spike protein with the chromatography media. The virus recovery relates solely to the chromatography step, and not to the overall process yield, which includes viral loss during the clarification and UF/DF steps, as described above. CC700 combines size exclusion (700 kDa cutoff) and binding chromatography using an octylamine ligand that is both hydrophobic and positively charged [65]. CC700 does not require binding and elution of the virus, as rVSV-S cannot enter the inner pores of the resin and is collected in the flow-through. Thus, as opposed to ion-exchange chromatography, purification with CC700 does not require high salt conditions and is ideal for virus purification and contaminant removal. The use of this technique for purification of the rVSV-S virus resulted in viral infectivity above 85% and efficient removal of host cell proteins (>99% clearance). The high virus recovery is attributed to the mild conditions required for purification with this resin. The binding capacity of the CC700 column to adsorb Vero HCP at a 1% breakthrough was determined to be 0.69 mg/mL, yielding an overall HCP clearance of 99% (DBC_1%_), underscoring its importance in upscaling the downstream process. The binding capacity was determined by loading the cell harvest directly to the column before the clarification and ultrafiltration step, which removed many of the HCPs. This further emphasizes the high binding capacity of the column to enable HCP clearance of large volumes of feed streams. Indeed, when directly applying the clarified harvest following the Denarase treatment to a 100 mL column without an initial ultrafiltration step, the recovery remained high, and regulatory demands for HCP removal were achieved. The use of the CC700 column is further advantageous, as it enables a rapid and robust, one-step purification platform for the rVSV-S-based vaccine. This is particularly valuable when considering scale-up. Moreover, this one-step purification scheme can be applied directly following the clarification step and does not require an initial ultrafiltration step.

## 5. Conclusions

This study reports a robust, efficient, high-throughput method for downstream purification of an rVSV-S vaccine candidate for COVID-19. In contrast to ion-exchange chromatography techniques, which exhibited low product recovery, the use of Capto^TM^ Core 700 resin resulted in high virus recovery as well as satisfactory removal of HCP contaminants.

## Figures and Tables

**Table 1:** Summary of rVSV-S recovery following purification using membrane adsorbers and packed-bed chromatography.

Chromatography Method	Mode	Column/Membrane	NaCl Concentration (mM)	Recovery (%)
Membrane adsorbers	Strong anion exchange	Mustang^®^ Q	150	13
		Natrix^®^ Q	150	14
		QF5	150	3
	Weak anion exchange	DF5	150	2
Packed-bed	Strong anion exchange	HiTrap Q XL	100	32
		HiTrap Q XL	150	33
		Fractogel^®^ TMEA	150	18
	Weak anion exchange	Fractogel^®^ DMEA	50	27
		Fractogel^®^ DMEA	100	26
		Fractogel^®^ DMEA	150	26
Mixed-mode	Size-exclusion and anion-exchange	Capto^TM^ Core 700	150	85

**Table 2:** Purification of rVSV-S on a 1 mL Q-XL anion exchanger with 150 mM NaCl. Fractions of 1 mL were collected, united in accordance with conductivity levels and analyzed for virus recovery and HCP.

Sample	Description	Total rVSV-S (PFU)	Recovery (%)	Total HCP (µg)
1	Source	4.0 × 10^8^	-	694
2	Flow-through	4.6 × 10^6^	1	256
3	Elution 16–45 ms/cm	4.4 × 10^7^	11	241
4	Elution 46–60 ms/cm	4.2 × 10^7^	11	3.97
5	Elution 61–80 ms/cm	8.4 × 10^7^	21	9.72

## Data Availability

The data presented in this study are available on request from the corresponding authors.
